# 5-Diethyl­amino-2-[(*E*)-(4-methyl-3-nitro­phenyl)­imino­meth­yl]phenol: a redetermination

**DOI:** 10.1107/S1600536809001731

**Published:** 2009-01-17

**Authors:** Hoong-Kun Fun, Reza Kia, A. M. Vijesh, Arun M. Isloor

**Affiliations:** a X-ray Crystallography Unit, School of Physics, Universiti Sains Malaysia, 11800 USM, Penang, Malaysia; b SeQuent Scientific Limited, No. 120 A&B, Industrial Area, Baikampady, New Mangalore, Karnataka 575 011, India; c Department of Chemistry, National Institute of Technology-Karnataka, Surathkal, Mangalore 575 025, India

## Abstract

The title compound, C_18_H_21_N_3_O_3_, is a potential bidentate Schiff base ligand. The whole mol­ecule is disordered with a refined site-occupancy ratio of 0.567 (4):0.433 (4) and not just one ethyl group as reported previously [Sarojini *et al.* (2007 ▶). *Acta Cryst*. E**63**, o4782–o4782]. Using the whole mol­ecule disorder, *R* values are much smaller than those published. An intra­molecular O—H⋯N hydrogen bond generates a six-membered ring, producing an *S*(6) ring motif. The dihedral angle between the mean plane of the two benzene rings (major component) is 9.0 (5)°. The crystal structure shows short C⋯C [3.189 (15)–3.298 (12) Å] and C⋯O [2.983 (5)–3.149 (13) Å] contacts. Inter­molecular C—H⋯O inter­actions link neighbouring mol­ecules into dimers with *R*_2_^2^(18) motifs. In the crystal structure, these dimers are linked together by inter­molecular C—H⋯O inter­actions into one-dimensional extended chains along the *b* axis. The crystal structure is further stabilized by inter­molecular π–π stacking inter­actions [centroid–centroid distances = 3.458 (8)–3.691 (6) Å].

## Related literature

For the previous determination of this structure, see: Sarojini *et al.* (2007 ▶). For details of hydrogen-bond motifs, see: Bernstein *et al.* (1995 ▶). For the application of Schiff bases in synthesis, coordination chemistry and biomedical activities, see: Patai (1970 ▶); Tai *et al.* (2003 ▶); Ittel *et al.* (2000 ▶); Kabeer *et al.* (2001 ▶); Pandeya *et al.* (1999 ▶); More *et al.* (2001 ▶); Singh & Dash (1988 ▶); Isloor *et al.* (2009 ▶); Pathak *et al.* (2000 ▶); Vazzanaa *et al.* (2004 ▶); Samadhiya & Halve (2001 ▶); Aydoğan *et al.* (2001 ▶).
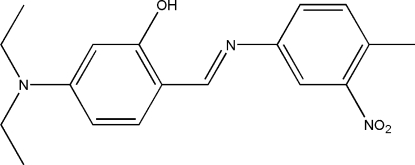


## Experimental

### 

#### Crystal data

C_18_H_21_N_3_O_3_
*M* *_r_* = 327.38
Monoclinic, 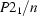

*a* = 7.2777 (1) Å
*b* = 22.1792 (5) Å
*c* = 10.3473 (2) Å
β = 103.713 (1)°
*V* = 1622.59 (5) Å^3^
*Z* = 4
Mo *K*α radiation
μ = 0.09 mm^−1^
*T* = 100.0 (1) K
0.49 × 0.25 × 0.04 mm

#### Data collection

Bruker SMART APEXII CCD area-detector diffractometer
Absorption correction: multi-scan (*SADABS*; Bruker, 2005 ▶) *T*_min_ = 0.956, *T*_max_ = 0.997
21678 measured reflections
4744 independent reflections
3239 reflections with *I* > 2σ(*I*)
*R*_int_ = 0.037

#### Refinement

*R*[*F* ^2^ > 2σ(*F* ^2^)] = 0.043
*wR*(*F* ^2^) = 0.117
*S* = 1.02
4744 reflections
412 parameters
1197 restraints
H-atom parameters constrained
Δρ_max_ = 0.21 e Å^−3^
Δρ_min_ = −0.22 e Å^−3^

### 

Data collection: *APEX2* (Bruker, 2005 ▶); cell refinement: *SAINT* (Bruker, 2005 ▶); data reduction: *SAINT*; program(s) used to solve structure: *SHELXTL* (Sheldrick, 2008 ▶); program(s) used to refine structure: *SHELXTL*; molecular graphics: *SHELXTL*; software used to prepare material for publication: *SHELXTL* and *PLATON* (Spek, 2003 ▶).

## Supplementary Material

Crystal structure: contains datablocks global, I. DOI: 10.1107/S1600536809001731/at2709sup1.cif

Structure factors: contains datablocks I. DOI: 10.1107/S1600536809001731/at2709Isup2.hkl

Additional supplementary materials:  crystallographic information; 3D view; checkCIF report

## Figures and Tables

**Table 1:** Hydrogen-bond geometry (Å, °)

*D*—H⋯*A*	*D*—H	H⋯*A*	*D*⋯*A*	*D*—H⋯*A*
O1*A*—H1*A*⋯N1*A*	0.84	1.95	2.68 (2)	144
C12*A*—H12*A*⋯O1*A*^i^	0.95	2.53	3.329 (19)	141
C15*A*—H15*A*⋯O3*A*^ii^	0.98	2.26	2.983 (5)	130

Symmetry codes: (i) 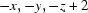; (ii) 
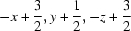
.

**Fig. 1.**
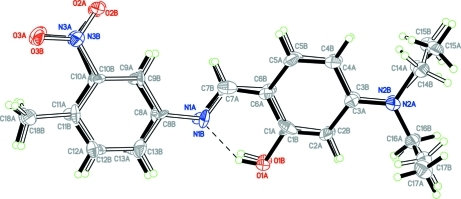
The molecular structure of (I), showing 50% probability displacement ellipsoids and the atomic numbering. Intramolecular hydrogen bond is shown as a dashed line. Open bonds show the minor component.

**Fig. 2.**
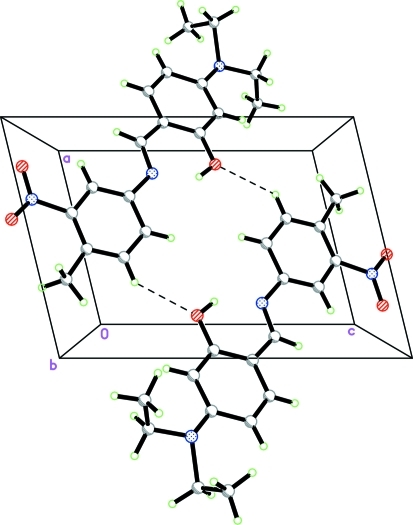
The crystal packing of the major component of (I), viewed down the b-axis, showing dimer formation by a R22(18) motif.

**Fig. 3.**
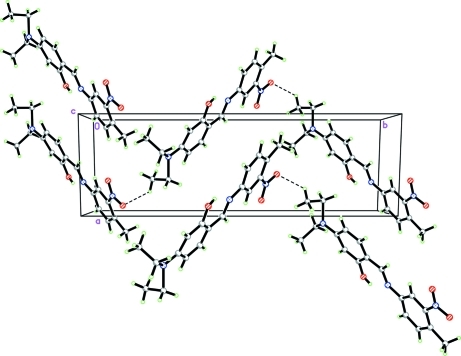
The crystal packing of the major component of (I), viewed down the c-axis, showing 1-D extended chain along the b-axis.

## supplementary crystallographic information

### Comment

Compounds with the structure of –C═N– (azomethine group) are known as Schiff bases, which are usually synthesized by the condensation of primary amines and active carbonyl groups. The chemistry of the carbon-nitrogen double bond plays a vital role in the progresses of chemistry (Patai 1970). They have been used as intermediates in medical substrates and as ligands in complex formation with some metal ions (Tai *et al.*, 2003). Recently multi-dentate complexes of iron and nickel showed high activities of ethylene oligomerization and polymerization (Ittel *et al.*, 2000). They have shown biological activities including antibacterial (Kabeer *et al.*, 2001; Pandeya *et al.*, 1999), antifungal (More *et al.*, 2001; Singh & Dash 1988), anticancer (Isloor *et al.*, 2009; Pathak *et al.*, 2000), anti-inflammatory (Vazzanaa *et al.*, 2004) and herbicidal (Samadhiya & Halve 2001) activities. In addition, Schiff bases have also been used as starting materials in the synthesis of large bioactive and industrial compounds via ring closure, cycloaddition and replacement reactions (Aydoğan *et al.*, 2001).

In the title compound, (I), (Fig. 1), an intramolecular O—H···N hydrogen bond generate a six-membered ring, producing *S*(6) ring motif (Table 1). Intermolecular C—H···O interactions link neighbouring molecules into dimers with *R_2_^2^(18)* motifs (Table 1, Fig. 2). The whole molecule is disordered with a site occupancy ratio of 0.567 (4)/0.433 (4) and not just the ethyl group as mentioned in the previously reported article (Sarojini *et al.*, 2007). Using the whole molecule disorder, R-values are much smaller than those published.

The two substituted benzene rings are not coplanar and make a dihedral angle of 9.0 (5)° (for major component A). The interesting features of the crystal structure is short C1A···C8A^i^ [3.298 (12) Å;(i) 1-x,-y,2-z], C1B···C8B^i^ [3.329 (16) Å], C3B···C12B^i^ [3.189 (15) Å], C15A···O3A^ii^ [2.983 (5) Å; (ii) 3/2-x,-1/2+y,3/2-z], C8B···O3B^iii^ [3.149 Å; (iii) -x,-y,1-z], and C13B···O3B^iii^ [3.116 (15) Å] contacts which are shorter than the sum of the van der Waals radii of these atoms. In the crystal structure, these dimers are linked together by intermolecular C—H···O interaction into 1-D extended chains along the *b*-axis (Fig. 3). The crystal structure is further stabilized by weak intermolecular π-π interactions [*Cg*1··· *Cg*2^i^ = 3.7744 (6) Å; *Cg*2···*Cg*3^i^ = 3.473 (7) Å; *Cg*3···*Cg*4^i^ = 3.458 (8) Å and *Cg*1···*Cg*4 = 3.670 (7) Å: *Cg*1, *Cg*2, *Cg*3, and *Cg*4 are the centroids of the C1A–C6A, C8A–C13A, C1B–C6B, and C8B–C13B benzene rings].

### Experimental

An equimolar mixture of 4-(diethylamino)-2-hydroxybenzaldehyde (0.5 g, 2.59 mmol) and 4-methyl-3-nitro aniline (0.393 g, 2.59 mmol) in ethanol (10 mL) were refluxed for 4 h. Catalytic amount of sulfuric acid was also added. The precipitated solid was filtered and recrystallised from acetone to yield orange coloured crystalline solid [0.41 g, 82 %, m.p 405 K]).

### Refinement

H atoms of the hydroxy groups were positioned by a freely rotating O—H bond and constrained with a fixed distance of 0.84 Å. The rest of the hydrogen atoms were positioned geometrically and refined using a riding model with C—H = 0.95–0.99 Å and U_iso_(H) = 1.2 or 1.5 U_eq_(C). A rotating-group model was applied for the methyl hydrogen atoms of the methoxy groups. Rigid bond, similarity and simulation restraints were applied.

### Crystal data

**Table:**

C_18_H_21_N_3_O_3_	*F*(000) = 696
*M**_r_* = 327.38	*D*_x_ = 1.340 Mg m^−^^3^
Monoclinic, *P*2_1_/*n*	Melting point: 405 K
Hall symbol: -P 2yn	Mo *K*α radiation, λ = 0.71073 Å
*a* = 7.2777 (1) Å	Cell parameters from 4554 reflections
*b* = 22.1792 (5) Å	θ = 2.7–29.8°
*c* = 10.3473 (2) Å	µ = 0.09 mm^−^^1^
β = 103.713 (1)°	*T* = 100 K
*V* = 1622.59 (5) Å^3^	Plate, yellow
*Z* = 4	0.49 × 0.25 × 0.04 mm

### Data collection

**Table:**

Bruker SMART APEXII CCD area-detector diffractometer	4744 independent reflections
Radiation source: fine-focus sealed tube	3239 reflections with *I* > 2σ(*I*)
graphite	*R*_int_ = 0.037
φ and ω scans	θ_max_ = 30.1°, θ_min_ = 2.2°
Absorption correction: multi-scan (*SADABS*; Bruker, 2005)	*h* = −10→10
*T*_min_ = 0.956, *T*_max_ = 0.997	*k* = −31→31
21678 measured reflections	*l* = −14→14

### Refinement

**Table:**

Refinement on *F*^2^	Primary atom site location: structure-invariant direct methods
Least-squares matrix: full	Secondary atom site location: difference Fourier map
*R*[*F*^2^ > 2σ(*F*^2^)] = 0.043	Hydrogen site location: inferred from neighbouring sites
*wR*(*F*^2^) = 0.117	H-atom parameters constrained
*S* = 1.02	*w* = 1/[σ^2^(*F*_o_^2^) + (0.0561*P*)^2^ + 0.1724*P*] where *P* = (*F*_o_^2^ + 2*F*_c_^2^)/3
4744 reflections	(Δ/σ)_max_ < 0.001
412 parameters	Δρ_max_ = 0.21 e Å^−^^3^
1197 restraints	Δρ_min_ = −0.22 e Å^−^^3^

### Special details

**Table:**

Experimental. The low-temperature data was collected with the Oxford Cyrosystem Cobra low-temperature attachment.
Geometry. All esds (except the esd in the dihedral angle between two l.s. planes) are estimated using the full covariance matrix. The cell esds are taken into account individually in the estimation of esds in distances, angles and torsion angles; correlations between esds in cell parameters are only used when they are defined by crystal symmetry. An approximate (isotropic) treatment of cell esds is used for estimating esds involving l.s. planes.
Refinement. Refinement of F^2^ against ALL reflections. The weighted R-factor wR and goodness of fit S are based on F^2^, conventional R-factors R are based on F, with F set to zero for negative F^2^. The threshold expression of F^2^ > 2sigma(F^2^) is used only for calculating R-factors(gt) etc. and is not relevant to the choice of reflections for refinement. R-factors based on F^2^ are statistically about twice as large as those based on F, and R- factors based on ALL data will be even larger.

### Fractional atomic coordinates and isotropic or equivalent isotropic displacement parameters (Å^2^)

**Table:**

	*x*	*y*	*z*	*U*_iso_*/*U*_eq_	Occ. (<1)
O1A	0.3739 (19)	0.0973 (10)	1.0926 (17)	0.0234 (9)	0.567 (4)
H1A	0.3089	0.0791	1.0262	0.035*	0.567 (4)
C1A	0.5374 (9)	0.1157 (3)	1.0663 (5)	0.0125 (7)	0.567 (4)
C2A	0.647 (2)	0.1575 (12)	1.1497 (19)	0.0255 (9)	0.567 (4)
H2AA	0.6140	0.1682	1.2301	0.031*	0.567 (4)
C3A	0.8077 (5)	0.18510 (16)	1.1189 (3)	0.0255 (6)	0.567 (4)
C4A	0.8620 (5)	0.16478 (18)	1.0018 (4)	0.0296 (7)	0.567 (4)
H4AA	0.9726	0.1803	0.9803	0.035*	0.567 (4)
C5A	0.7531 (7)	0.1227 (3)	0.9210 (6)	0.0252 (10)	0.567 (4)
H5AA	0.7911	0.1099	0.8436	0.030*	0.567 (4)
C6A	0.5879 (14)	0.0974 (8)	0.9470 (12)	0.0197 (10)	0.567 (4)
C7A	0.4817 (12)	0.0550 (5)	0.8538 (8)	0.0179 (13)	0.567 (4)
H7AA	0.5300	0.0422	0.7807	0.021*	0.567 (4)
N1A	0.3211 (11)	0.0336 (5)	0.8673 (8)	0.0179 (9)	0.567 (4)
C8A	0.2039 (12)	−0.0048 (5)	0.7729 (8)	0.0250 (14)	0.567 (4)
C9A	0.2462 (14)	−0.0271 (7)	0.6580 (10)	0.0230 (14)	0.567 (4)
H9AA	0.3631	−0.0171	0.6376	0.028*	0.567 (4)
C10A	0.1184 (11)	−0.0637 (5)	0.5730 (8)	0.0221 (11)	0.567 (4)
C11A	−0.0553 (11)	−0.0821 (6)	0.5965 (9)	0.0226 (11)	0.567 (4)
C12A	−0.0955 (14)	−0.0564 (5)	0.7096 (9)	0.0309 (12)	0.567 (4)
H12A	−0.2169	−0.0637	0.7256	0.037*	0.567 (4)
C13A	0.0291 (11)	−0.0210 (5)	0.8002 (9)	0.0264 (12)	0.567 (4)
H13A	−0.0024	−0.0078	0.8796	0.032*	0.567 (4)
N2A	0.9119 (3)	0.22760 (14)	1.1993 (3)	0.0402 (7)	0.567 (4)
C14A	1.1461 (3)	0.22808 (9)	1.21715 (19)	0.0262 (5)	0.567 (4)
H14A	1.2168	0.2369	1.3093	0.031*	0.567 (4)
H14B	1.1942	0.1908	1.1843	0.031*	0.567 (4)
C15A	1.1392 (3)	0.28153 (10)	1.1233 (2)	0.0327 (6)	0.567 (4)
H15A	1.2677	0.2912	1.1158	0.049*	0.567 (4)
H15B	1.0847	0.3165	1.1585	0.049*	0.567 (4)
H15C	1.0609	0.2712	1.0353	0.049*	0.567 (4)
C16A	0.8713 (9)	0.2448 (3)	1.3260 (6)	0.0268 (11)	0.567 (4)
H16A	0.9900	0.2580	1.3881	0.032*	0.567 (4)
H16B	0.8232	0.2092	1.3655	0.032*	0.567 (4)
C17A	0.7284 (9)	0.2947 (3)	1.3107 (9)	0.0343 (11)	0.567 (4)
H17A	0.7058	0.3047	1.3979	0.052*	0.567 (4)
H17B	0.6097	0.2816	1.2509	0.052*	0.567 (4)
H17C	0.7764	0.3304	1.2734	0.052*	0.567 (4)
C18A	−0.1980 (12)	−0.1223 (4)	0.5104 (10)	0.0286 (13)	0.567 (4)
H18A	−0.3071	−0.1270	0.5498	0.043*	0.567 (4)
H18B	−0.1412	−0.1619	0.5034	0.043*	0.567 (4)
H18C	−0.2394	−0.1045	0.4216	0.043*	0.567 (4)
N3A	0.184 (3)	−0.0827 (19)	0.455 (2)	0.0313 (14)	0.567 (4)
O2A	0.3440 (11)	−0.0694 (3)	0.4470 (8)	0.0529 (17)	0.567 (4)
O3A	0.0868 (6)	−0.1188 (2)	0.3802 (5)	0.0700 (13)	0.567 (4)
O1B	0.398 (3)	0.0963 (13)	1.092 (2)	0.0234 (9)	0.433 (4)
H1B	0.3551	0.0680	1.0391	0.035*	0.433 (4)
C1B	0.5589 (14)	0.1191 (5)	1.0618 (9)	0.0306 (17)	0.433 (4)
C2B	0.665 (3)	0.1575 (15)	1.155 (2)	0.0255 (9)	0.433 (4)
H2BA	0.6148	0.1741	1.2235	0.031*	0.433 (4)
C3B	0.8515 (5)	0.17175 (19)	1.1452 (4)	0.0181 (7)	0.433 (4)
C4B	0.9079 (6)	0.1517 (2)	1.0301 (4)	0.0218 (8)	0.433 (4)
H4BA	1.0277	0.1636	1.0172	0.026*	0.433 (4)
C5B	0.7946 (8)	0.1158 (4)	0.9378 (8)	0.0221 (11)	0.433 (4)
H5BA	0.8374	0.1034	0.8620	0.027*	0.433 (4)
C6B	0.6180 (19)	0.0966 (10)	0.9512 (17)	0.0197 (10)	0.433 (4)
C7B	0.4953 (17)	0.0590 (7)	0.8598 (12)	0.030 (2)	0.433 (4)
H7BA	0.5334	0.0463	0.7824	0.036*	0.433 (4)
N1B	0.3323 (17)	0.0409 (7)	0.8762 (12)	0.0250 (18)	0.433 (4)
C8B	0.2194 (14)	0.0014 (6)	0.7836 (10)	0.0169 (13)	0.433 (4)
C9B	0.2642 (19)	−0.0211 (9)	0.6684 (14)	0.0189 (13)	0.433 (4)
H9BA	0.3822	−0.0117	0.6491	0.023*	0.433 (4)
C10B	0.1362 (14)	−0.0571 (6)	0.5834 (10)	0.0213 (14)	0.433 (4)
C11B	−0.0420 (16)	−0.0732 (8)	0.6030 (12)	0.028 (2)	0.433 (4)
C12B	−0.0749 (16)	−0.0544 (7)	0.7258 (11)	0.0330 (19)	0.433 (4)
H12B	−0.1823	−0.0688	0.7536	0.040*	0.433 (4)
C13B	0.0489 (16)	−0.0153 (7)	0.8050 (13)	0.033 (2)	0.433 (4)
H13B	0.0131	0.0013	0.8800	0.040*	0.433 (4)
N2B	0.9676 (3)	0.20711 (11)	1.2388 (2)	0.0158 (5)	0.433 (4)
C14B	1.0549 (4)	0.26562 (12)	1.1520 (3)	0.0288 (7)	0.433 (4)
H14C	1.0015	0.2654	1.0547	0.035*	0.433 (4)
H14D	1.0482	0.3063	1.1899	0.035*	0.433 (4)
C15B	1.2453 (4)	0.23619 (13)	1.1946 (3)	0.0343 (8)	0.433 (4)
H15D	1.3381	0.2593	1.1602	0.051*	0.433 (4)
H15E	1.2384	0.1950	1.1595	0.051*	0.433 (4)
H15F	1.2839	0.2350	1.2920	0.051*	0.433 (4)
C16B	0.9026 (12)	0.2355 (5)	1.3477 (7)	0.0245 (12)	0.433 (4)
H16C	1.0144	0.2490	1.4162	0.029*	0.433 (4)
H16D	0.8356	0.2050	1.3891	0.029*	0.433 (4)
C17B	0.7722 (11)	0.2891 (4)	1.3056 (11)	0.0315 (13)	0.433 (4)
H17D	0.7339	0.3054	1.3832	0.047*	0.433 (4)
H17E	0.6598	0.2761	1.2389	0.047*	0.433 (4)
H17F	0.8388	0.3203	1.2674	0.047*	0.433 (4)
C18B	−0.1932 (17)	−0.1105 (6)	0.5125 (13)	0.0300 (17)	0.433 (4)
H18D	−0.3026	−0.1143	0.5518	0.045*	0.433 (4)
H18E	−0.1429	−0.1507	0.5014	0.045*	0.433 (4)
H18F	−0.2320	−0.0907	0.4256	0.045*	0.433 (4)
N3B	0.185 (4)	−0.078 (2)	0.459 (3)	0.0313 (14)	0.433 (4)
O2B	0.3391 (12)	−0.0633 (4)	0.4405 (10)	0.0260 (11)	0.433 (4)
O3B	0.0621 (6)	−0.10006 (19)	0.3677 (5)	0.0255 (7)	0.433 (4)

### Atomic displacement parameters (Å^2^)

**Table:**

	*U*^11^	*U*^22^	*U*^33^	*U*^12^	*U*^13^	*U*^23^
O1A	0.020 (3)	0.0286 (7)	0.0251 (4)	−0.003 (2)	0.0111 (17)	−0.0049 (4)
C1A	0.0190 (15)	0.0127 (15)	0.0082 (12)	0.0006 (11)	0.0079 (10)	−0.0015 (10)
C2A	0.031 (2)	0.0267 (5)	0.0230 (12)	−0.0060 (19)	0.0144 (18)	−0.0060 (8)
C3A	0.0251 (15)	0.0271 (17)	0.0258 (16)	−0.0008 (11)	0.0091 (11)	−0.0056 (12)
C4A	0.0284 (16)	0.034 (2)	0.0314 (18)	−0.0034 (12)	0.0175 (13)	−0.0041 (13)
C5A	0.026 (2)	0.0303 (18)	0.0211 (16)	0.0044 (15)	0.0082 (14)	−0.0067 (14)
C6A	0.019 (3)	0.0228 (6)	0.0184 (8)	0.003 (2)	0.0072 (19)	0.0009 (7)
C7A	0.027 (2)	0.0150 (18)	0.0087 (19)	0.0030 (15)	−0.0020 (15)	−0.0034 (16)
N1A	0.0219 (19)	0.018 (2)	0.0152 (18)	0.0029 (12)	0.0063 (15)	−0.0020 (14)
C8A	0.0211 (17)	0.024 (2)	0.024 (2)	0.0017 (13)	−0.0067 (14)	−0.0046 (14)
C9A	0.020 (3)	0.035 (4)	0.015 (2)	0.0024 (18)	0.005 (2)	−0.0029 (15)
C10A	0.0218 (18)	0.024 (2)	0.0150 (15)	0.0008 (14)	−0.0058 (11)	−0.0054 (13)
C11A	0.019 (2)	0.030 (3)	0.020 (2)	0.0001 (15)	0.0067 (16)	−0.0031 (14)
C12A	0.0195 (16)	0.043 (2)	0.026 (2)	−0.0028 (14)	−0.0026 (13)	−0.0072 (17)
C13A	0.0192 (16)	0.039 (3)	0.026 (3)	0.0015 (15)	0.0150 (19)	−0.0062 (17)
N2A	0.0351 (12)	0.0537 (17)	0.0389 (14)	−0.0199 (11)	0.0227 (10)	−0.0212 (12)
C14A	0.0232 (10)	0.0282 (10)	0.0292 (10)	−0.0032 (8)	0.0105 (8)	−0.0030 (8)
C15A	0.0344 (11)	0.0308 (11)	0.0390 (11)	−0.0074 (9)	0.0208 (9)	0.0020 (9)
C16A	0.023 (2)	0.033 (3)	0.0247 (19)	−0.0049 (15)	0.0064 (15)	−0.0040 (16)
C17A	0.032 (3)	0.0292 (18)	0.0424 (18)	0.0006 (16)	0.0093 (18)	0.0078 (14)
C18A	0.0211 (15)	0.030 (3)	0.0303 (18)	−0.0061 (16)	−0.0038 (12)	−0.0059 (18)
N3A	0.0284 (5)	0.044 (5)	0.0211 (13)	−0.0034 (11)	0.0058 (7)	−0.0080 (7)
O2A	0.028 (2)	0.092 (4)	0.040 (3)	−0.001 (2)	0.0118 (18)	−0.028 (3)
O3A	0.083 (3)	0.087 (3)	0.052 (2)	−0.0527 (19)	0.0408 (18)	−0.047 (2)
O1B	0.020 (3)	0.0286 (7)	0.0251 (4)	−0.003 (2)	0.0111 (17)	−0.0049 (4)
C1B	0.023 (2)	0.029 (3)	0.043 (3)	−0.0032 (18)	0.0160 (18)	0.0165 (19)
C2B	0.031 (2)	0.0267 (5)	0.0230 (12)	−0.0060 (19)	0.0144 (18)	−0.0060 (8)
C3B	0.0189 (16)	0.0160 (16)	0.0188 (16)	0.0090 (10)	0.0033 (11)	0.0086 (11)
C4B	0.0216 (17)	0.0228 (18)	0.0221 (17)	0.0055 (12)	0.0072 (12)	0.0046 (12)
C5B	0.017 (2)	0.029 (2)	0.024 (2)	0.0043 (18)	0.0121 (18)	0.0053 (15)
C6B	0.019 (3)	0.0228 (6)	0.0184 (8)	0.003 (2)	0.0072 (19)	0.0009 (7)
C7B	0.031 (3)	0.031 (4)	0.032 (5)	0.007 (2)	0.017 (3)	0.009 (3)
N1B	0.028 (2)	0.020 (3)	0.021 (2)	0.0010 (16)	−0.0066 (16)	−0.0077 (16)
C8B	0.019 (3)	0.025 (3)	0.0084 (19)	0.0045 (18)	0.0057 (19)	−0.0035 (16)
C9B	0.0133 (18)	0.021 (2)	0.018 (2)	−0.0030 (15)	−0.0052 (15)	−0.0053 (17)
C10B	0.016 (2)	0.036 (4)	0.014 (2)	−0.0026 (17)	0.008 (2)	−0.0050 (18)
C11B	0.023 (2)	0.029 (4)	0.026 (2)	−0.0086 (18)	−0.0078 (17)	−0.0045 (19)
C12B	0.019 (3)	0.063 (4)	0.020 (3)	−0.003 (2)	0.011 (3)	−0.006 (2)
C13B	0.031 (3)	0.043 (4)	0.021 (3)	−0.001 (2)	−0.0029 (19)	−0.008 (3)
N2B	0.0172 (9)	0.0156 (11)	0.0132 (10)	0.0058 (7)	0.0011 (7)	0.0053 (8)
C14B	0.0297 (14)	0.0258 (14)	0.0327 (14)	−0.0003 (11)	0.0111 (11)	−0.0025 (11)
C15B	0.0268 (15)	0.0375 (16)	0.0411 (16)	−0.0004 (11)	0.0128 (12)	−0.0035 (12)
C16B	0.024 (3)	0.030 (3)	0.020 (2)	0.0050 (16)	0.0061 (18)	−0.0041 (19)
C17B	0.027 (3)	0.031 (2)	0.038 (2)	−0.003 (2)	0.009 (2)	−0.0073 (17)
C18B	0.033 (2)	0.027 (4)	0.032 (3)	−0.008 (2)	0.0111 (18)	−0.002 (2)
N3B	0.0284 (5)	0.044 (5)	0.0211 (13)	−0.0034 (11)	0.0058 (7)	−0.0080 (7)
O2B	0.023 (2)	0.0343 (19)	0.023 (2)	−0.0071 (15)	0.0100 (17)	−0.0035 (16)
O3B	0.0187 (9)	0.0372 (17)	0.0163 (10)	0.0021 (11)	−0.0043 (7)	−0.0078 (11)

### Geometric parameters (Å, °)

**Table:**

O1A—C1A	1.346 (7)	O1B—C1B	1.376 (9)
O1A—H1A	0.8400	O1B—H1B	0.8400
C1A—C2A	1.384 (7)	C1B—C2B	1.377 (10)
C1A—C6A	1.427 (7)	C1B—C6B	1.406 (9)
C2A—C3A	1.420 (8)	C2B—C3B	1.418 (10)
C2A—H2AA	0.9500	C2B—H2BA	0.9500
C3A—N2A	1.363 (4)	C3B—N2B	1.371 (5)
C3A—C4A	1.433 (5)	C3B—C4B	1.420 (6)
C4A—C5A	1.373 (6)	C4B—C5B	1.361 (8)
C4A—H4AA	0.9500	C4B—H4BA	0.9500
C5A—C6A	1.409 (7)	C5B—C6B	1.391 (8)
C5A—H5AA	0.9500	C5B—H5BA	0.9500
C6A—C7A	1.436 (7)	C6B—C7B	1.409 (9)
C7A—N1A	1.299 (7)	C7B—N1B	1.301 (9)
C7A—H7AA	0.9500	C7B—H7BA	0.9500
N1A—C8A	1.419 (7)	N1B—C8B	1.410 (9)
C8A—C9A	1.388 (7)	C8B—C13B	1.363 (9)
C8A—C13A	1.413 (7)	C8B—C9B	1.401 (9)
C9A—C10A	1.383 (7)	C9B—C10B	1.375 (9)
C9A—H9AA	0.9500	C9B—H9BA	0.9500
C10A—C11A	1.403 (6)	C10B—C11B	1.405 (8)
C10A—N3A	1.474 (7)	C10B—N3B	1.484 (9)
C11A—C12A	1.394 (7)	C11B—C12B	1.410 (9)
C11A—C18A	1.493 (7)	C11B—C18B	1.512 (9)
C12A—C13A	1.384 (7)	C12B—C13B	1.373 (9)
C12A—H12A	0.9500	C12B—H12B	0.9500
C13A—H13A	0.9500	C13B—H13B	0.9500
N2A—C16A	1.462 (6)	N2B—C16B	1.463 (8)
N2A—C14A	1.670 (3)	N2B—C14B	1.779 (4)
C14A—C15A	1.526 (3)	C14B—C15B	1.501 (4)
C14A—H14A	0.9900	C14B—H14C	0.9900
C14A—H14B	0.9900	C14B—H14D	0.9900
C15A—H15A	0.9800	C15B—H15D	0.9800
C15A—H15B	0.9800	C15B—H15E	0.9800
C15A—H15C	0.9800	C15B—H15F	0.9800
C16A—C17A	1.501 (6)	C16B—C17B	1.518 (8)
C16A—H16A	0.9900	C16B—H16C	0.9900
C16A—H16B	0.9900	C16B—H16D	0.9900
C17A—H17A	0.9800	C17B—H17D	0.9800
C17A—H17B	0.9800	C17B—H17E	0.9800
C17A—H17C	0.9800	C17B—H17F	0.9800
C18A—H18A	0.9800	C18B—H18D	0.9800
C18A—H18B	0.9800	C18B—H18E	0.9800
C18A—H18C	0.9800	C18B—H18F	0.9800
N3A—O3A	1.219 (10)	N3B—O2B	1.225 (9)
N3A—O2A	1.220 (7)	N3B—O3B	1.242 (13)
			
O1A—C1A—C2A	119.0 (8)	O1B—C1B—C2B	115.9 (10)
O1A—C1A—C6A	120.7 (7)	O1B—C1B—C6B	119.6 (10)
C2A—C1A—C6A	120.0 (6)	C2B—C1B—C6B	123.9 (9)
C1A—C2A—C3A	122.2 (8)	C1B—C2B—C3B	118.5 (10)
C1A—C2A—H2AA	118.9	C1B—C2B—H2BA	120.7
C3A—C2A—H2AA	118.9	C3B—C2B—H2BA	120.7
N2A—C3A—C2A	121.9 (4)	N2B—C3B—C2B	121.2 (6)
N2A—C3A—C4A	120.6 (3)	N2B—C3B—C4B	121.3 (4)
C2A—C3A—C4A	117.4 (5)	C2B—C3B—C4B	117.3 (6)
C5A—C4A—C3A	119.6 (4)	C5B—C4B—C3B	121.6 (5)
C5A—C4A—H4AA	120.2	C5B—C4B—H4BA	119.2
C3A—C4A—H4AA	120.2	C3B—C4B—H4BA	119.2
C4A—C5A—C6A	123.3 (5)	C4B—C5B—C6B	122.0 (7)
C4A—C5A—H5AA	118.4	C4B—C5B—H5BA	119.0
C6A—C5A—H5AA	118.4	C6B—C5B—H5BA	119.0
C5A—C6A—C1A	117.3 (6)	C5B—C6B—C1B	116.1 (8)
C5A—C6A—C7A	118.7 (6)	C5B—C6B—C7B	124.6 (9)
C1A—C6A—C7A	124.0 (6)	C1B—C6B—C7B	119.2 (8)
N1A—C7A—C6A	121.5 (7)	N1B—C7B—C6B	123.2 (10)
N1A—C7A—H7AA	119.3	N1B—C7B—H7BA	118.4
C6A—C7A—H7AA	119.3	C6B—C7B—H7BA	118.4
C7A—N1A—C8A	123.7 (6)	C7B—N1B—C8B	120.4 (9)
C9A—C8A—C13A	118.3 (6)	C13B—C8B—C9B	116.5 (8)
C9A—C8A—N1A	125.7 (6)	C13B—C8B—N1B	117.7 (8)
C13A—C8A—N1A	116.0 (6)	C9B—C8B—N1B	125.8 (8)
C10A—C9A—C8A	120.1 (6)	C10B—C9B—C8B	119.5 (9)
C10A—C9A—H9AA	119.9	C10B—C9B—H9BA	120.2
C8A—C9A—H9AA	119.9	C8B—C9B—H9BA	120.2
C9A—C10A—C11A	124.0 (6)	C9B—C10B—C11B	124.3 (7)
C9A—C10A—N3A	113.1 (6)	C9B—C10B—N3B	117.9 (7)
C11A—C10A—N3A	123.0 (6)	C11B—C10B—N3B	117.8 (8)
C12A—C11A—C10A	113.8 (6)	C10B—C11B—C12B	114.5 (7)
C12A—C11A—C18A	119.4 (6)	C10B—C11B—C18B	127.6 (9)
C10A—C11A—C18A	126.7 (6)	C12B—C11B—C18B	117.8 (9)
C13A—C12A—C11A	124.5 (7)	C13B—C12B—C11B	119.8 (8)
C13A—C12A—H12A	117.7	C13B—C12B—H12B	120.1
C11A—C12A—H12A	117.7	C11B—C12B—H12B	120.1
C12A—C13A—C8A	119.1 (6)	C8B—C13B—C12B	124.6 (9)
C12A—C13A—H13A	120.5	C8B—C13B—H13B	117.7
C8A—C13A—H13A	120.5	C12B—C13B—H13B	117.7
C3A—N2A—C16A	121.8 (4)	C3B—N2B—C16B	122.2 (5)
C3A—N2A—C14A	118.5 (2)	C3B—N2B—C14B	107.1 (2)
C16A—N2A—C14A	108.5 (3)	C16B—N2B—C14B	107.1 (5)
C15A—C14A—N2A	93.22 (16)	C15B—C14B—N2B	87.1 (2)
C15A—C14A—H14A	113.1	C15B—C14B—H14C	114.1
N2A—C14A—H14A	113.1	N2B—C14B—H14C	114.1
C15A—C14A—H14B	113.1	C15B—C14B—H14D	114.1
N2A—C14A—H14B	113.1	N2B—C14B—H14D	114.1
H14A—C14A—H14B	110.5	H14C—C14B—H14D	111.3
C14A—C15A—H15A	109.5	C14B—C15B—H15D	109.5
C14A—C15A—H15B	109.5	C14B—C15B—H15E	109.5
H15A—C15A—H15B	109.5	H15D—C15B—H15E	109.5
C14A—C15A—H15C	109.5	C14B—C15B—H15F	109.5
H15A—C15A—H15C	109.5	H15D—C15B—H15F	109.5
H15B—C15A—H15C	109.5	H15E—C15B—H15F	109.5
N2A—C16A—C17A	112.4 (4)	N2B—C16B—C17B	114.2 (6)
N2A—C16A—H16A	109.1	N2B—C16B—H16C	108.7
C17A—C16A—H16A	109.1	C17B—C16B—H16C	108.7
N2A—C16A—H16B	109.1	N2B—C16B—H16D	108.7
C17A—C16A—H16B	109.1	C17B—C16B—H16D	108.7
H16A—C16A—H16B	107.9	H16C—C16B—H16D	107.6
C16A—C17A—H17A	109.5	C16B—C17B—H17D	109.5
C16A—C17A—H17B	109.5	C16B—C17B—H17E	109.5
H17A—C17A—H17B	109.5	H17D—C17B—H17E	109.5
C16A—C17A—H17C	109.5	C16B—C17B—H17F	109.5
H17A—C17A—H17C	109.5	H17D—C17B—H17F	109.5
H17B—C17A—H17C	109.5	H17E—C17B—H17F	109.5
C11A—C18A—H18A	109.5	C11B—C18B—H18D	109.5
C11A—C18A—H18B	109.5	C11B—C18B—H18E	109.5
H18A—C18A—H18B	109.5	H18D—C18B—H18E	109.5
C11A—C18A—H18C	109.5	C11B—C18B—H18F	109.5
H18A—C18A—H18C	109.5	H18D—C18B—H18F	109.5
H18B—C18A—H18C	109.5	H18E—C18B—H18F	109.5
O3A—N3A—O2A	122.4 (11)	O2B—N3B—O3B	120.2 (13)
O3A—N3A—C10A	117.3 (8)	O2B—N3B—C10B	118.2 (9)
O2A—N3A—C10A	119.3 (7)	O3B—N3B—C10B	120.4 (11)
C1B—O1B—H1B	109.5		
			
O1A—C1A—C2A—C3A	−171 (2)	O1B—C1B—C2B—C3B	166 (3)
C6A—C1A—C2A—C3A	2(3)	C6B—C1B—C2B—C3B	−5(4)
C1A—C2A—C3A—N2A	178.0 (16)	C1B—C2B—C3B—N2B	−176 (2)
C1A—C2A—C3A—C4A	−5(3)	C1B—C2B—C3B—C4B	8(4)
N2A—C3A—C4A—C5A	−178.9 (4)	N2B—C3B—C4B—C5B	178.9 (4)
C2A—C3A—C4A—C5A	3.6 (15)	C2B—C3B—C4B—C5B	−6(2)
C3A—C4A—C5A—C6A	−0.3 (12)	C3B—C4B—C5B—C6B	−0.2 (16)
C4A—C5A—C6A—C1A	−2(2)	C4B—C5B—C6B—C1B	3(3)
C4A—C5A—C6A—C7A	178.3 (10)	C4B—C5B—C6B—C7B	−179.4 (17)
O1A—C1A—C6A—C5A	174.7 (16)	O1B—C1B—C6B—C5B	−172 (2)
C2A—C1A—C6A—C5A	1(2)	C2B—C1B—C6B—C5B	0(4)
O1A—C1A—C6A—C7A	−6(2)	O1B—C1B—C6B—C7B	11 (3)
C2A—C1A—C6A—C7A	−179 (2)	C2B—C1B—C6B—C7B	−178 (3)
C5A—C6A—C7A—N1A	−174.8 (13)	C5B—C6B—C7B—N1B	179 (2)
C1A—C6A—C7A—N1A	6(2)	C1B—C6B—C7B—N1B	−4(3)
C6A—C7A—N1A—C8A	175.0 (14)	C6B—C7B—N1B—C8B	−177.5 (19)
C7A—N1A—C8A—C9A	3(2)	C7B—N1B—C8B—C13B	−179.1 (17)
C7A—N1A—C8A—C13A	−176.6 (12)	C7B—N1B—C8B—C9B	−1(3)
C13A—C8A—C9A—C10A	0(2)	C13B—C8B—C9B—C10B	1(3)
N1A—C8A—C9A—C10A	−179.3 (13)	N1B—C8B—C9B—C10B	−176.9 (17)
C8A—C9A—C10A—C11A	−2(2)	C8B—C9B—C10B—C11B	1(3)
C8A—C9A—C10A—N3A	179 (2)	C8B—C9B—C10B—N3B	177 (3)
C9A—C10A—C11A—C12A	4.5 (19)	C9B—C10B—C11B—C12B	−6(3)
N3A—C10A—C11A—C12A	−177 (2)	N3B—C10B—C11B—C12B	177 (3)
C9A—C10A—C11A—C18A	−178.9 (13)	C9B—C10B—C11B—C18B	177.7 (18)
N3A—C10A—C11A—C18A	0(3)	N3B—C10B—C11B—C18B	1(4)
C10A—C11A—C12A—C13A	−6.6 (19)	C10B—C11B—C12B—C13B	10 (2)
C18A—C11A—C12A—C13A	176.6 (12)	C18B—C11B—C12B—C13B	−173.2 (15)
C11A—C12A—C13A—C8A	6(2)	C9B—C8B—C13B—C12B	3(3)
C9A—C8A—C13A—C12A	−2.3 (19)	N1B—C8B—C13B—C12B	−178.4 (16)
N1A—C8A—C13A—C12A	177.4 (11)	C11B—C12B—C13B—C8B	−9(3)
C2A—C3A—N2A—C16A	3.6 (16)	C2B—C3B—N2B—C16B	−4(2)
C4A—C3A—N2A—C16A	−173.8 (3)	C4B—C3B—N2B—C16B	170.9 (5)
C2A—C3A—N2A—C14A	143.3 (15)	C2B—C3B—N2B—C14B	−128 (2)
C4A—C3A—N2A—C14A	−34.1 (4)	C4B—C3B—N2B—C14B	47.0 (3)
C3A—N2A—C14A—C15A	102.7 (3)	C3B—N2B—C14B—C15B	−111.2 (2)
C16A—N2A—C14A—C15A	−112.8 (4)	C16B—N2B—C14B—C15B	116.1 (4)
C3A—N2A—C16A—C17A	−88.1 (6)	C3B—N2B—C16B—C17B	−74.0 (9)
C14A—N2A—C16A—C17A	128.8 (5)	C14B—N2B—C16B—C17B	49.9 (8)
C9A—C10A—N3A—O3A	175 (3)	C9B—C10B—N3B—O2B	2(6)
C11A—C10A—N3A—O3A	−4(5)	C11B—C10B—N3B—O2B	179 (4)
C9A—C10A—N3A—O2A	5(5)	C9B—C10B—N3B—O3B	−166 (4)
C11A—C10A—N3A—O2A	−174 (3)	C11B—C10B—N3B—O3B	11 (6)

### Hydrogen-bond geometry (Å, °)

**Table:**

*D*—H···*A*	*D*—H	H···*A*	*D*···*A*	*D*—H···*A*
O1A—H1A···N1A	0.84	1.95	2.68 (2)	144
C12A—H12A···O1A^i^	0.95	2.53	3.329 (19)	141
C15A—H15A···O3A^ii^	0.98	2.26	2.983 (5)	130

Symmetry codes: (i) −*x*, −*y*, −*z*+2; (ii) −*x*+3/2, *y*+1/2, −*z*+3/2.
